# Personal Exposure to Ultrafine Particles and Oxidative DNA Damage

**DOI:** 10.1289/ehp.7562

**Published:** 2005-05-31

**Authors:** Peter S. Vinzents, Peter Møller, Mette Sørensen, Lisbeth E. Knudsen, Ole Hertel, Finn Palmgren Jensen, Bente Schibye, Steffen Loft

**Affiliations:** 1 Department of Occupational and Environmental Health, University of Copenhagen, Copenhagen, Denmark; 2 National Environmental Research Institute, Copenhagen, Denmark; 3 National Institute of Occupational Health, Copenhagen, Denmark

**Keywords:** comet assay, exposure, oxidative DNA damage, personal, traffic, ultrafine particles

## Abstract

Exposure to ultrafine particles (UFPs) from vehicle exhaust has been related to risk of cardiovascular and pulmonary disease and cancer, even though exposure assessment is difficult. We studied personal exposure in terms of number concentrations of UFPs in the breathing zone, using portable instruments in six 18-hr periods in 15 healthy nonsmoking subjects. Exposure contrasts of outdoor pollution were achieved by bicycling in traffic for 5 days and in the laboratory for 1 day. Oxidative DNA damage was assessed as strand breaks and oxidized purines in mononuclear cells isolated from venous blood the morning after exposure measurement. Cumulated outdoor and cumulated indoor exposures to UFPs each were independent significant predictors of the level of purine oxidation in DNA but not of strand breaks. Ambient air concentrations of particulate matter with an aero-dynamic diameter of ≤10 μm (PM_10_), nitrous oxide, nitrogen dioxide, carbon monoxide, and/or number concentration of UFPs at urban background or busy street monitoring stations was not a significant predictor of DNA damage, although personal UFP exposure was correlated with urban background concentrations of CO and NO_2_, particularly during bicycling in traffic. The results indicate that biologic effects of UFPs occur at modest exposure, such as that occurring in traffic, which supports the relationship of UFPs and the adverse health effects of air pollution.

Epidemiologic studies have associated exposure to ambient air particulate matter (PM) with pulmonary and cardiovascular diseases and cancer (Brunekreef and Holgate 2002; Pope et al. 2002). To date, the majority of studies have dealt with the relationship between health outcomes and the ambient levels of PM_10_ and PM_2.5_, which are the mass of particles with a aerodynamic diameters ≤10 and 2.5 μm, respectively. Recently, however, interest has focused on the ultrafine particle (UFP) fraction with a diameter ≤100 nm, which are abundant in numbers but contribute little to particle mass. Mechanistically, UFPs are important because of the adverse health effects caused by their high alveolar deposition fraction, large surface area, chemical composition, ability to induce inflammation, and potential to translocate to the circulation (Donaldson et al. 2001; Donaldson and Tran 2002; Nemmar et al. 2002, 2004; Schins et al. 2004). Vehicle emissions, particularly those related to diesel engines, are a major source of ambient UFPs, which penetrate to the indoor environment (Franck et al. 2003; Levy et al. 2002).

A few epidemiologic studies have associated daily changes in number concentrations of UFPs measured at a single urban background monitoring station with daily cardiovascular and pulmonary mortality as well as lung function or use of medicine among patients with asthma (Ibald-Mulli et al. 2002; Penttinen et al. 2001; Peters et al. 1997; von Klot et al. 2002; Wichmann et al. 2000). However, the relationship between number concentrations in urban background and personal exposure to UFPs is not known, and direct links between ambient UFPs and health effects have not been established. Because people spend around 90% of their time indoors (Jenkins et al. 1992), it is widely recognized that a significant proportion of personal exposure to particles occurs in the indoor environment. Indoor UFPs consist of a combination of ambient particles that readily penetrate buildings and infiltrate indoor air (Franck et al. 2003; Levy et al. 2002; Long et al. 2001a; Ozkaynak et al. 1996) and nonambient particles generated indoors during the daily activities of home occupants. Major indoor sources of UFPs include smoking, cooking, candle burning, and other combustion-related processes as well as chemical reactions between, for example, terpenes and ozone (Abt et al. 2000; Dennekamp et al. 2001; Levy et al. 2000; Long et al. 2000; Ozkaynak et al. 1996).

Personal monitors can be used to measure individual exposure. By means of biomarkers based on putative mechanisms of action, exposure can be related to biologic effects, allowing substantiation of causal relationships and identification of relevant sources and exposure scenarios. The mechanisms of action of adverse health effects of PM are based on experimental studies thought to involve induction of inflammation and oxidative stress (Donaldson et al. 2001; Donaldson and Tran 2002; Knaapen et al. 2004; Schins et al. 2004). The generation of oxidative stress may involve radicals and soluble transition metals on the surface of UFPs and activation of production of reactive oxygen species in macrophages, granulocytes, and target cells as well as redox cycling of quinone metabolites of polyaromatic hydrocarbons. In this context UFPs appear more potent than fine or coarse particles per unit mass (Brown et al. 2000, 2001). Experimental studies *in vivo* and *in vitro* point to DNA oxidation as an important target of UFPs and fine-fraction PM (Brown et al. 2000, 2001; Dybdahl et al. 2003; Knaapen et al. 2004; Risom et al. 2003a; Schins et al. 2002). Recently, we have shown significant relationships between individual exposure to PM_2.5_, assessed as mass collected on filters over 48 hr, and biomarkers of oxidative damage to DNA bases in terms of 8-oxodeoxyguanosine (8-oxodG), proteins, and lipids among healthy subjects (Sørensen et al. 2003a, 2003b, 2003c). However, this exposure measurement cannot discriminate between indoor and out-door exposure, and ambient PM_2.5_ mass is influenced by long-range transport of nitrate-and sulfate-based fine particles (Ruuskanen et al. 2001).

Because UFPs are ubiquitous, even in indoor environments, exposure is unavoidable, and only levels of exposure can be compared. In the present cross-over study, time-resolved personal exposure to traffic-and indoor-related UFPs was assessed by portable equipment and related to oxidative DNA damage in mononuclear blood cells on 6 different days in 15 subjects after low-intensity bicycling exercise in traffic or indoors. Measurements with outdoor bicycling were repeated on 5 days in order to have variation in outdoor exposure for each individual due to differences in traffic density and meteorologic conditions. The control of outdoor exposure and the wide gradient for each subject allowed study of dose–response relationship and comparison of the contribution of outdoor exposure and indoor exposure. We also assessed personal exposure and DNA damage in relation to ambient concentrations of air pollutants measured at two curbside monitoring stations on busy streets and at one urban background station.

## Materials and Methods

### Personal monitoring.

Fifteen healthy non-smoking subjects, 10 males (25.3 ± 3.5 mean years of age, ± SD) and 5 females (25.4 ± 1.5 years) participated in the study after giving informed consent. The local ethics committee approved the study. In a cross-over design with subjects serving as their own control, personal exposure to UFPs was measured for 18 hr on weekdays six times for each person in the period from March through June 2003. Two subjects were studied simultaneously on each occasion. Condensation particle counters (TSI 3007; TSI, St. Paul, MN, USA) with continuous measurement of the number concentrations of UFPs (10–100 nm) were carried in backpacks with the inlet tube placed in the breathing zone. The instruments were equipped with external batteries, and the subjects were trained to supply them with 2-propanol every 8 hr. The instruments count particles optically after they have grown in size in an atmosphere saturated with 2-propanol, which must be supplied at 8-hr intervals. Time series of 1 min average concentrations were logged during each day. For practical reasons the sampling was interrupted during the night. Two data sets were lost because of technical errors. Exposure was referred to as number concentration of UFPs per milliliter. Cumulated exposure was defined as average concentration multiplied by time with minute as time unit; that is, the unit of cumulated exposure was minutes × UFPs per milliliter (for convenience, tables and figures display units of 10^6^ minutes × UFPs/mL). The particle counters were validated by showing strong correlations in measurements (both instruments: *r* > 0.999, *n* = 13) when compared with a TSI 3010 stationary particle counter (TSI) on aerosols of NaCl in 10–20 nm size ranges from 20 to 200 nm and the regression lines going through the origin. Comparison of the two employed TSI 3007 instruments showed a constant difference in counting efficiency of 8.9%, which was corrected for. With this correction the two instruments also gave identical results when carried by different subjects that bicycled the exposure route together (data not shown).

Five of the 6 days of exposure measurement included bicycling in central Copenhagen on a 20-km predefined route during morning and/or afternoon rush hours. The mean bicycling time was 93 ± 15 min. This allowed study of dose–response relationships associated with variations in outdoor exposure for each individual due to differences in traffic density and meteorologic conditions. One exposure measurement day included the same workload at the same intensity on an ergometer bicycle in a room at the Panum Institute (Copenhagen, Denmark) with air intake away from streets and minimal number concentrations of UFPs.

The relationship between heart rate and workload was established for each subject from an ergometer bicycle test, and the average workloads during traffic bicycling on each of the 5 days were calculated from the average heart rates measured during traffic bicycling. Increased pulmonary ventilation will increase the deposition possibility of UFPs dependent on the breathing pattern (Daigle et al. 2003). A conservative estimate is achieved by assuming proportionality between increased pulmonary ventilation and increased deposition (*D*) of UFPs. Because pulmonary ventilation during moderate dynamic exercise increases linearly with work intensity (*P*), the increased UFP deposition during traffic bicycling compared with UFP deposition during rest or light exercise (*P* = 60 W ) can be found as:


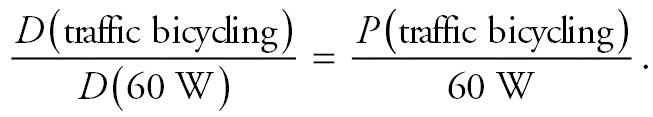


Individual values of increased pulmonary deposition of UFPs during traffic bicycling were estimated, and cumulated personal traffic exposure was adjusted. The average estimated increase in deposition was a factor of 1.43 ± 0.37) (*n* = 67). The subjects kept a diary for recording periods of bicycling, other outdoor activities, and indoor time and activities, including exposure to cooking fumes, burning candles, and environmental tobacco smoke. The subjects were asked to keep the latter exposures at the lowest possible level. The distribution of time spent on outdoor and indoor activities is shown in Table 1.

### Determination of oxidative DNA damage.

In the morning after each exposure measurement day, mononuclear blood cells were isolated from venous blood samples in Vacutainer CPT tubes with sodium heparin tubes (Becton Dickinson and Company, Rutherford, NJ, USA) and centrifuged at 1,650 × *g* for 20 min at room temperature. The cell layer was obtained and washed in cold RPMI medium from Gibco (Grand Island, NY, USA) and centrifuged at 400 × *g* for 15 min at 4°C. Most of the supernatant was removed, and the pellet was resuspended in cold preservation medium with volume-percent as follows: 40% RPMI, 50% fetal bovine serum (Gibco), and 10% dimethyl sulfoxide (AppliChem, Darmstadt, Germany). The samples were stored at −80°C for later analysis. Oxidative DNA damage was determined by single-cell gel electrophoresis (comet assay) as strand breaks and base damage in terms of sites sensitive to formamidopyrimidine glycosylase (FPG), which cleaves DNA at sites of oxidized purines and mainly detects 8-oxodG (Collins et al. 1997; Sørensen et al. 2003d). Briefly, cells were thawed on ice, embedded in 0.75% low-melting-point agarose (Sigma, Copenhagen, Denmark) on Gelbond films (BioWhittaker Molecular Applications, Rockland, ME, USA), and lysed for a minimum of 1 hr at 4°C (2.5 M NaCl; 0.1 M EDTA; 10 mM Tris, base pH 10; 1% Triton X-100). The gel-embedded nuclei were washed 3 × 5 min in cold buffer (40 mM HEPES, 0.1 M KCl, 0.5 mM EDTA, 0.2 mg/mL bovine serum albumin, pH 8) to remove the lysis solution. The FPG-sensitive sites were detected by incubation of the agarose-embedded nuclei with 1 μg/mL FPG protein (kindly provided by A. Collins, University of Olso, Oslo, Sweden) for 45 min at 37°C. The nuclei were subsequently treated in alkaline solution (300 mM NaOH, 1 mM EDTA, pH > 13) for 40 min and electrophoresed in the same solution at 4°C for 20 min at 25 V and 300 mA. The level of DNA damage in each sample was scored in 100 nuclei according to a five-class system (range of score is 0–400). The net level of FPG-sensitive sites was obtained as the difference in score between samples incubated with FPG protein and buffer. This score was translated into lesions per 10^6^ base pairs (bp) by means of a calibration curve based on induction of strand breaks by X ray, which has a known yield [European Standards Committee on Oxidative DNA Damage (ESCODD) 2003; Møller et al. 2004a]. We have used a conversion factor of 0.056 Gy equivalents per score, or 0.011 lesions/10^6^ bp per score (assuming that a human diploid cell contains 4 × 10^12^ Da DNA, corresponding to 6 × 10^9^ bp). All samples from a subject were coded and analyzed simultaneously in duplicate, minimizing effects of interassay variation. The method has been validated in interlaboratory trials and is believed to be free from artifactual oxidation problems (ESCODD 2003).

### Fixed station monitoring.

Ambient concentrations of air pollutants were measured on all exposure days at two curbside busy street stations along the bicycling route and at one urban background station on a rooftop at 20 m height approximately 500 m from the start and end of the bicycling route. Ambient air concentrations of nitric oxide, nitrogen dioxide, carbon monoxide, and PM_10_ were measured continuously with a 1-hr time resolution by standard methods at all stations under the Danish Air Quality Monitoring Programme (Kemp and Palmgren 2004). The instruments used for PM_10_ measurements were the tapered element oscillating microbalance (series 1400a ambient particulate monitor; Rupprecht & Patashnick, East Greenbush, NY, USA). The instrument was approved by the U.S. Environmental Protection Agency for PM_10_ ambient particulate monitoring. The mass measurements were performed at 50°C to stabilize the water content of the particles, but at the same time other volatile compounds, for example, ammonium nitrate and organic volatiles, will be lost. One street station also measured size-fractionated number concentrations of UFPs by a scanning mobility particle sizer (Palmgren et al. 2003). Temperature, relative humidity, and wind speed were recorded at the urban background station.

### Statistical analysis.

Statistical analysis of DNA damage was carried out by means of mixed-effects models, which allow both random and fixed effects. The subject level was a random factor, and cumulated exposure to UFPs occurring during bicycling, remaining time outdoors and indoors, and monitoring station values were tested as potential predictor variables with fixed effects. The effect of bicycling indoors or outdoors on total exposure to UFPs and DNA damage was also assessed by two-factorial analysis of variance, including subject as factor. The DNA damage and personal exposure variables were cubic root transformed before analysis to achieve normal distributions. Similarly, in another analysis the relationship between personal log-transformed exposure occurring outdoors during bicycling and other activities, or indoors, and 24-hr average monitoring station log-transformed measurements was analyzed by linear mixed-effects models with subject level as random factor. SPSS (version 11.0; SPSS Inc., Chicago, IL, USA) was used for analysis.

## Results

**Figure 1:**
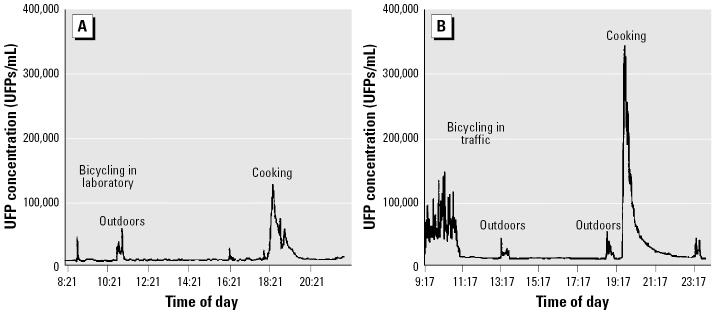
Examples of personal UFP exposure profiles on a laboratory (*A*) and traffic (*B*) bicycling day. Indoor and outdoor periods and activities are marked.

Typical 18-hr personal exposure profiles are shown in Figure 1. Peak concentration of indoor UFPs usually coincided with presence of indoor sources such as cooking, burning candles, or environmental tobacco smoke recorded in the subjects’ diaries. The exposure during bicycling in traffic was significantly inversely correlated with air temperature and wind speed as well as directly correlated with the measured concentrations of ambient pollutants at both background and street monitoring stations (Table 2**)**. Weaker but significant correlations were found between indoor UFP exposure and air temperature (inverse) and concentrations of NO_2_ (background station) and CO (background station and street station) and between UFP exposure during other outdoor activities and air temperature and CO concentrations (Table 2).

In linear mixed-effects models with subjects as a random factor, background monitoring station measurements of ambient temperature and CO concentration, and ambient temperature and NO_2_ concentration at one of the street stations were the only significant predictors of UFP exposure during bicycling in traffic (*R*^2^ = 0.60 and *R*^2^ = 0.74, respectively). In contrast, air temperature was the only significant predictor of UFP exposure during other outdoor activities (*R*^2^ = 0.09), and background concentration of CO was the only significant predictor of indoor UFP exposure (*R*^2^ = 0.11).

Bicycling in traffic increased the cumulated exposure to UFPs significantly, although indoor exposure contributed more because of the much longer time spent indoors (Table 3**)**.

**Figure 2:**
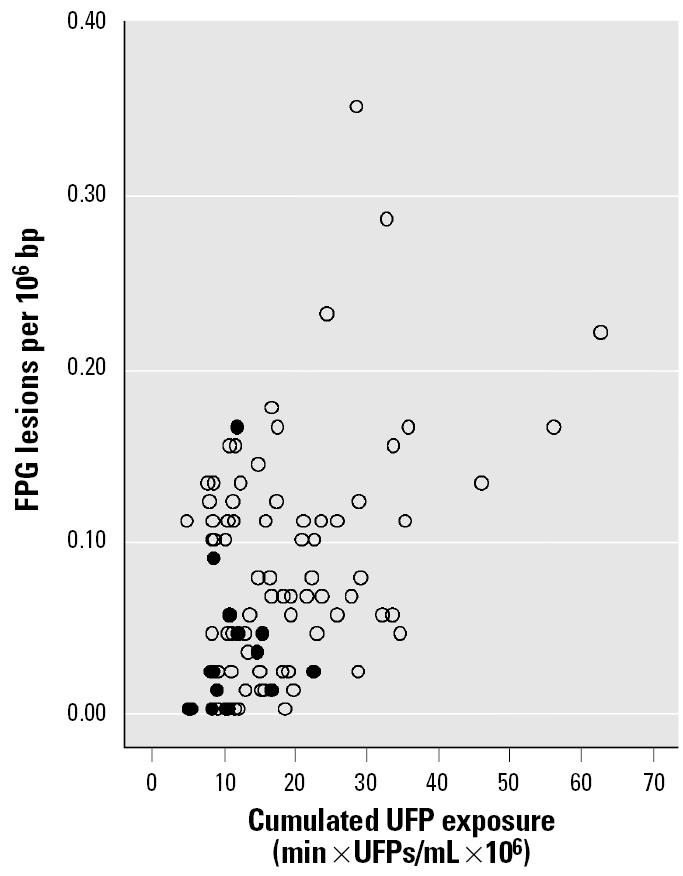
Relationship between oxidative DNA base damage as FPG lesions in mononuclear blood cells on the morning after exposure and exposure to UFPs during 5 days of bicycling in traffic (open circles) and 1 day of bicycling in the laboratory (solid circles) in 15 healthy subjects. One data point at (*x*, *y*) = (12 × 10^6^, 0.62) is omitted from the figure to limit the scale. Indoor and outdoor exposures to UFPs were significant independent predictors of the FPG lesions in a mixed-effects model (*R*^2^ = 0.503).

After bicycling in traffic the level of oxidative DNA base damage in terms of FPG-sensitive sites was increased 4-fold (*p* < 0.001) compared with the level measured after bicycling indoors, but there was no effect on DNA strand breaks (Table 3, Figure 2**)**. The level of FPG-sensitive sites (per 10^6^ bp) was significantly predicted by the personal cumulated exposure to UFPs with independent contributions from outdoor and indoor observation periods. The regression coefficients of the mixed-effects model of level of DNA damage, including both outdoor and indoor exposures, with subjects as random factor, were estimated as 1.50 × 10^−3^ [95% confidence interval (CI), 0.59 × 10^−3^ to 2.42 × 10^−3^; *p* = 0.002] for cumulated outdoor exposure and 1.07 × 10^−3^ (95% CI, 0.37 × 10^−3^ to 1.77 × 10^−3^; *p* = 0.003) for cumulated indoor exposure.

The level of DNA damage and the cumulated exposure were cubic root transformed before the mixed-effects model analysis. The model explained 50.3% (*R*^2^) of the variation, and the residuals were randomly and normally distributed as confirmed by nonparametric tests (Runs test and Kolmogorov-Smirnov test). The regression coefficient should in principle describe the dose–response relationship, although they are not easy to interpret in absolute numbers because of the cubic root transformations. The levels of DNA damage were not significantly associated with any 24-hr average concentration of ambient air pollutants measured at a monitoring station (Pearson’s *r* < 0.303).

## Discussion

In this study oxidative DNA base damage in circulating mononuclear blood cells was associated with personal exposure to UFPs, and short-term higher intensity exposure in traffic was associated with elevated levels of damage. Cumulated outdoor and indoor exposures contributed independently to the association, which showed clear dose–response relationships. The level of damage was not associated with ambient concentrations of air pollutants at a monitoring station, although the concentrations of several of these were associated with personal UFP exposure during bicycling, in particular.

Oxidative DNA damage is mutagenic and carcinogenic per se and may be considered a biomarker of oxidative stress, which is also thought to be involved in cardiovascular and pulmonary disease due to UFPs (Brown et al. 2001; Donaldson et al. 2001; Li et al. 2003; Schins et al. 2004). After indoor bicycling the level of DNA damage was very low and at a level corresponding to well-nourished healthy volunteers with minimum exposures (Møller and Loft 2004). This low level could be assessed with good precision by an X-ray–calibrated visual scoring system, which we find more sensitive than computer-based image analysis (Møller et al. 2004a). The increase in FPG-sensitive sites in DNA of median 0.06 per 10^6^ bp in circulating mononuclear cells after outdoor bicycling would require a radiation dose of approximately 0.14 Gy to induce, assuming a yield of 0.43 FPG sites per 10^6^ bp/Gy, as found in mice *in vivo* (Risom et al. 2003b). However, radiation induces many types of DNA damage, and this comparison cannot be used for risk characterization. We have previously found a significant association between oxidative DNA base damage, without changes in strand breaks, and personal exposure to PM in terms of PM_2.5_ measured as mass over 48 hr in young healthy subjects in Copenhagen (Sørensen et al. 2003b). In that study DNA damage was assessed at the end of the monitoring period, similar to the design in the present study. The lack of measurable effects of PM on DNA strand breaks may be due to the very rapid repair by ligases, whereas guanine oxidation is repaired relatively slowly by base excision followed by strand nicking, insertion of nucleotide(s) in the gap, and rejoining by ligases (Hoeijmakers 2001; Risom et al. 2003b). Indeed, DNA base oxidation has been found to be much more sensitive than strand breaks to environmental factors, including several types of air pollution, smoking, and antioxidant intervention (Avobge et al. 2005; Møller and Loft 2002, 2004; Møller et al. 2004b; Sørensen et al. 2003d). In a mouse study the level of oxidized guanine in lung DNA was increased, whereas strand breaks were unchanged 1 and 24 hr after inhalation of diesel exhaust particles (Risom et al. 2003a).

Similar to our findings for DNA base oxidation in the present and a previous study (Sørensen et al. 2003b), we have also found significant associations between personal exposure to black smoke, measured as reflectance of material collected on PM_2.5_ filters, and oxidation of plasma proteins, and a similar association between the mass of the filter material and lipid peroxidation in plasma, although the latter was significant only among women (Sørensen et al. 2003c). However, the cumulated exposure measurement in the previous studies did not allow assessment of effects of UFPs and distinction between outdoor and indoor sources (Sørensen et al. 2003b, 2003c). Staying outdoors in traffic, particularly during bicycling, provided higher intensity of exposure for limited periods of time, whereas staying indoors provided prolonged periods of generally low-intensity exposure, although with some activity-related peaks. Vehicle exhaust is the main source of outdoor UFPs, which can penetrate indoors where additional sources include environmental tobacco smoke, cooking, burning of candles, and chemical reactions (Abt et al. 2000; Dennekamp et al. 2001; Levy et al. 2000; Long et al. 2000; Ozkaynak et al. 1996). The parameter estimate of the mixed-effects model describing the level of DNA damage in relation to exposure to UFPs was nominally larger for outdoor than for indoor exposure. This could suggest larger potency of the outdoor UFPs, compared with indoor UFPs, possibly by a factor of 3 considering the cubic root transformations. The personal UFP monitors we used would also measure liquid droplets in the 10–100 nm size range, which could be particularly abundant during, for example, cooking and could have limited toxicologic potential. However, the 95% CIs had considerable overlap, and no firm conclusion can be drawn. Moreover, the particles we measured in numbers indoors or outdoors could not be characterized in other aspects that could have indicated causal components. Nevertheless, diesel exhaust particles have consistently been shown to induce 8-oxodG in experimental animals and *in vitro* (Brown et al. 2000, 2001; Dybdahl et al. 2003; Knaapen et al. 2004; Risom et al. 2003a; Schins et al. 2002). Moreover, UFPs can be translocated to the circulation upon inhalation and may interact directly with circulating mononuclear cells, possibly explaining the DNA base oxidation found in the present study (Donaldson et al. 2001; Donaldson and Tran 2002; Nemmar et al. 2002, 2004; Schins et al. 2004; Semmler et al. 2004). The toxicity of indoor particles has only been assessed for PM_2._ and coarse particles with respect to inflammatory potential *in vitro*, and the potential for inducing DNA damage is unknown, and indoor UFPs have yet to be investigated (Long et al. 2001b; Monn and Becker 1999; Roponen et al. 2003). Other studies with exposure assessment based on residence or occupation in urban areas also point to an association between ambient air pollution and oxidative DNA damage, for example, in nasal biopsies and leukocytes of subjects in Mexico City or in urine from bus drivers in Copenhagen (Calderon-Garciduenas et al. 1996, 1999; Fortoul et al. 2003, 2004; Loft et al. 1999)

Our subjects performed modest exercise in terms of bicycling at moderate speed. This increases internal exposure to UFPs by increasing both ventilation and probably lung deposition, as shown recently (Daigle et al. 2003). We took into account the increased ventilation in our exposure assessment by calculations based on the increases in heart rate at fixed workloads. Without this correction outdoor UFPs would have appeared even more potent with respect to induction of DNA base damage. We did not take into account a possible increase in the fractional deposition during outdoor bicycling caused by a change in the breathing pattern. This may also explain the possible higher potency of outdoor UFPs.

Personal exposure to UFPs when bicycling in traffic was inversely related to temperature and wind speed, which is consistent with increased formation through condensation of gases at low temperatures and dispersion by wind. Ambient concentration of UFPs and CO measured at street stations were the strongest predictors of outdoor personal UFP exposure during bicycling, which is consistent with traffic as the major source (Palmgren et al. 2003). Similarly, CO was the strongest predictor measured in urban background. The UFP exposure during other outdoor activities and indoor exposure were less strongly associated with 24-hr monitoring station measurements, and none of the monitoring station measurements was significantly associated with the level of oxidative DNA damage. Compared with direct measurement of personal exposure, monitoring stations measurements are poorer predictors of both exposure and biologic effects. Nevertheless, the significant association between CO concentrations in urban background and personal exposure to indoor UFPs supports that traffic emissions have some contribution to indoor UFPs.

This study design, including direct measurement of personal exposure and traffic-related contrasts, has proved promising in demonstrating association between UFPs and biologic effects in terms of oxidative DNA base damage. The results support the importance of UFPs in causing health effects related to generation of oxidative stress by air pollutants. Moreover, concern about the health effects of even small high-intensity exposures of UFPs in ambient air may be relevant.

## Figures and Tables

**Table 1:** Distribution of time (min) as mean ± SD spent in traffic, outdoors, and indoors on six occasions in each of 15 healthy subjects.

Bicycling (days)	Time bicycling on designated route	Time bicycling elsewhere	Time outdoors not bicycling	Time indoors
In traffic (*n* = 74)	93 ± 15	7 ± 21	62 ± 66	751 ± 65
Indoors (*n* = 14)	—	22 ± 21	59 ± 59	837 ± 62

**Table 2:** Geometric means (GM) and geometric SDs (GSD) of air pollutants concentrations, and partial correlation (subject controlled) between meteorologic conditions and ambient log-transformed concentrations of air pollutants measured as 24-hr averages at monitoring stations against personal exposure to UFPs for 15 subjects, each measured on five or six occasions.

Measure	GM (GSD) *n*^a^	Bicycling on exposure route (5 occasions)	Other outdoor activities (6 occasions)	Indoors (6 occasions)
UFPs (personal exposure)				
GM (GSD) *n*^a^	—	32.4^b^ (1.49) 74	19.6^b^ (1.78) 84	13.4^b^ (1.96) 89
Correlations				
Background station				
Temperature	—	−0.619*	−0.300*	−0.320*
Wind speed	—	−0.516*	−0.145	−0.132
NO_x_	13.4^c^ (1.61) 73	0.439*	0.207	0.259
NO_2_	11.3^c^ (1.52) 73	0.454*	0.237	0.293*
CO	273^c^ (1.35) 73	0.651*	0.317*	0.371*
PM_10_	16.9^c^ (1.53) 75	0.290	0.126	0.193
Street station 1				
UFPs	30.4^b^ (1.38) 75	0.493*	0.179	0.255
NO_x_	72.4^c^ (1.44) 72	0.486*	0.193	0.105
NO_2_	32.1^c^ (1.31) 72	0.394*	0.147	0.118
Street station 2				
NO_x_	51.7^c^ (1.76) 74	0.444*	0.228	0.226
NO_2_	24.2^c^ (1.49) 74	0.415*	0.207	0.266
CO	788^c^ (1.52) 74	0.556*	0.289*	0.311*
PM_10_	23.5^c^ (1.48) 75	0.428*	0.198	0.249

NO_*x*_, nitrogen oxide.

a GM (GSD) number of measurements.

b Data are expressed in units of 10^3^ UFPs/mL.

c Data are expressed as μg/m^3^.

* Significant correlations at the 0.01% level (two-tailed).

**Table 3:** Median and interquartile range of cumulated exposure to UFPs and oxidative DNA damage as FPG lesions and strand breaks (SB) in 15 subjects bicycling in traffic or indoors, on six occasions.

	Cumulated exposure to UFPs (10^6^ min × UFPs/mL)			DNA damage (per 10^6^ bp)	
Bicycling (days)	Traffic bicycling	Remaining time outdoors	Time indoors	FPG	SB
In traffic (*n* = 74)	3.01^a^ (2.25–4.44)	1.54^a^ (0.68–3.28)	10.5^a^ (5.86–16.7)	0.08^b^ (0.04–0.12)	0.06 (0.03–0.11)
Indoors (*n* = 14)	—	1.42 (0.52–2.41)	9.20 (6.15–13.1)	0.02 (0–0.04)	0.06 (0.02–0.12)

a Total UFP exposure (sum) increased compared with day with indoor bicycling (*p* = 0.004).

b DNA damage increased compared with day with indoor bicycling (*p* = 0.0003).
